# Evaluating the Effects of Cigarette Smoking and Heated Tobacco Products on Hard Dental Tissues: A Comparative Histological and Colorimetric Analysis

**DOI:** 10.1002/cre2.941

**Published:** 2024-08-05

**Authors:** Mahmoud Al Ankily, Fatma Makkeyah, Mahmoud M. Bakr, Mohamed Shamel

**Affiliations:** ^1^ Oral Biology Department, Faculty of Dentistry The British University in Egypt Cairo Egypt; ^2^ Fixed Prosthodontics Department, Faculty of Dentistry The British University in Egypt Cairo Egypt; ^3^ School of Medicine and Dentistry Griffith University Gold Coast Queensland Australia

**Keywords:** cementum, cigarette smoking, discoloration, enamel, heated tobacco, SEM

## Abstract

**Objectives:**

This study aimed to evaluate and compare the impact of cigarette smoking (CS) and heated tobacco (HT) on the alteration of color and ultrastructural characteristics of human enamel and cementum.

**Background:**

According to tobacco companies, a less harmful substitute for CS is HT products. Nevertheless, comprehensive research on the effects of HT on tooth structures has been lacking. This study aimed to evaluate and compare the impact of CS and HT on the alteration of color and ultrastructural characteristics of human enamel and cementum.

**Materials and Methods:**

Thirty intact and noncarious human maxillary premolars extracted for orthodontic treatment purposes, previously disinfected, were used in the study. The specimens were randomly separated into six groups (*n* = 10), as follows: Group 1: enamel without smoking exposure; Group 2: enamel exposed to CS; Group 3: enamel exposed to HT; Group 4: cementum without smoking exposure; Group 5: cementum exposed to CS; and Group 6: cementum exposed to HT. The measurement of color change was conducted using a spectrophotometer. The surface alterations and mineral composition of enamel and cementum were evaluated using scanning electron microscopy and energy‐dispersive X‐ray spectroscopy. ANOVA test followed by Tukey's post hoc test was used to determine significant differences between groups.

**Results:**

Results showed that CS had a more pronounced effect on enamel and cementum color changes than HT. The impact of CS and HT on color changes was more evident in cementum than in enamel. Surface morphology of enamel and cementum showed alterations in histology following exposure to both smoking types. Moreover, the mineral content experienced a significant reduction after using CS and HT. The reduction in calcium content after CS and HT exposure was similar. However, HT led to a significant decrease in the phosphorus content of enamel when compared with CS. At the same time, CS exposure in cementum resulted in a more significant reduction in Ca/P ratio than HT.

**Conclusions:**

Although HT may appear to present a lower danger to hard dental tissues than CS, it is not entirely harmless. CS results in more color changes on the enamel and cementum of teeth. Both smoking methods affected the mineral content of teeth, with CS having a significant effect on the roots, while HT significantly affected the crowns' mineral composition.

## Clinical Relevance

The study addresses a knowledge gap on how smoking and HT products affect hard tooth tissues. Few studies compared the histological and colorimetric effects of these two smoking strategies before this study. Our research shows that cigarette smoke and HT products affect dental enamel and cementum differently, revealing their potential tooth harm. The use of HT products may cause tooth cavities and periodontal disease. This research can help clinicians advise patients on the oral health effects of these smoking alternatives. This will help improve preventive and corrective dental procedures as HT products become more prevalent.

## 1 Introduction

Tobacco consumption is a global public health concern, as more than a billion people of different ethnic and socioeconomic statuses smoke across the globe, with a high prevalence of tobacco consumption among low‐ and middle‐income countries (Neuberger 2021; Le Foll et al. 2022). Despite its known harmful impacts, tobacco consumption continues to be one of the most common health‐related phenomena worldwide, leading to the mortality of millions annually, as its use is related to a broad spectrum of health‐related complications, for example, lung cancer, cardiovascular diseases, respiratory diseases, and oral health issues ranging from potentially malignant lesions to malignant life‐threatening complications (Varghese and Muntode Gharde 2023), so there is a great importance to shed some light on the associations between different methods of tobacco smoking and oral health (Komar et al. 2018).

Although cigarette smoking (CS) is one of the most common habits with adverse health consequences, tobacco companies' advertising strategies have contributed to its prevalent use (Nazir et al. 2019; Owens, Ha, and Soulakova 2019). In recent years, tobacco companies have advertised new alternatives to traditional combusted products. These alternatives include electronic cigarettes (e‐cigarettes) and heated tobacco (HT) products (Saravia and Pisinger 2020). These products were advertised as a clean and safe alternative to burnt tobacco. Thus, they not only gained the attention of young adults and adolescents but also encouraged nonsmokers to smoke, creating a nicotine‐addicted population (Kim and Friedman 2022).

There are variations in product design and mechanisms within these products; however, both deliver nicotine without burning tobacco. The e‐cigarettes are mainly formed of liquids containing nicotine that generate an inhalable nicotine aerosol when heated without containing tobacco (Chen, Zhuang, and Zhu 2016), which is different from HT products that will always contain tobacco. It is important to note that in HT products the tobacco is not lit directly. The temperature applied to the tobacco varies between ambient temperature and 350°C, depending on the apparatus (Vukas et al. 2023). HT products produce less side stream smoke and less toxic combustion products than heated cigarettes, thus resulting in the reduction of chronic diseases linked to traditional smoking, such as lung cancer, ischemic heart diseases, and chronic obstructive pulmonary diseases (Znyk, Jurewicz, and Kaleta 2021). The long‐term risk of these alternatives is still controversial, as the principal harmful compounds are present in HT emissions (Upadhyay et al. 2023; Ratajczak et al. 2020). New dangers continue to present themselves as the consumption of these alternative smoking methods continues to spread among young adults and adolescents (Znyk, Jurewicz, and Kaleta 2021; Luca et al. 2023). The efficacy of their assistance in facilitating smoking cessation remains mainly uncertain, and their influence on the initiation of smoking among young individuals is a subject of debate (Czoli et al. 2020). Thus, the influence of HT on smoking prevalence among the public remains uncertain. Understanding the dental health impacts of different tobacco consumption methods is essential (Camoni et al. 2023).

Tobacco use, whether through CS or HT, is responsible for a range of dental health issues. It leads to the discoloration of teeth and restorations, more plaque and calculus accumulation, which compromises tooth aesthetics (Karanjkar et al. 2023; Kurachi et al. 2023); it also leads to a more common incidence of implant failure, post‐extraction dry socket, and impaired socket healing owing to the vasoconstrictor effect of nicotine (Nazir et al. 2019; Owens, Ha, and Soulakova 2019). In addition, both forms of tobacco are associated with gum disease and periodontal disease, with CS compromising gum health, increasing the total number of gram‐negative anaerobes due to chronic tissue hypoxia produced by nicotine, reduction of tissue blood flow, lowered immune responses to infections, increased release of inflammatory mediators due to the frequent exposure to the harmful and potentially harmful products as a result of smoking, and impaired healing of the gum (Silva 2021; Zhang et al. 2019). This is associated with symptoms including bleeding, edematous gums, tooth mobility, gum recession, and the possibility of tooth loss if left untreated (Pouly et al. 2021; Yoshioka and Tabuchi 2021). Furthermore, tobacco consumption is closely associated with mucosal lesions and oral premalignant lesions (Komar et al. 2018). Moreover, CS is the most common cause of oral squamous cell carcinoma, which accounts for the most common type of oral cancer, affecting various oral regions and often carrying a poor prognosis (Jiang 2019). Moreover, smoking leads to xerostomia, decreasing salivary production and diminishing saliva's self‐cleaning and acid‐buffering capabilities, thereby increasing tooth decay (Kakoei et al. 2021; Rad et al. 2010). These dental health implications underline the importance of individual awareness of the associated complications of smoking and seeking support for smoking cessation to safeguard their oral health (Aljubran et al. 2022).

CS and HT products harm tooth enamel through various mechanisms, contributing to dental health problems. CS exposes individuals to numerous harmful chemicals, including tar and nicotine (Onor et al. 2017; Zhao et al. 2020). Tar adheres to enamel, causing staining, whereas nicotine contributes to yellowing (Karanjkar et al. 2023; Zanetti et al. 2019). Over time, these substances accumulate, significantly darkening enamel and impacting not only an individual's self‐confidence but also serving as a visible sign of the harmful effects of smoking on dental health (Calzada et al. 2021).

Similarly, HT products, though perceived as a potentially less harmful alternative to traditional cigarettes, can also lead to enamel discoloration (Haiduc et al. 2020). Their aerosol carries various chemicals, including flavoring agents and potentially toxic compounds like formaldehyde and acetaldehyde. These substances can gradually change color in contact with dental enamel, leading to staining and discoloration (Kurachi et al. 2023). Furthermore, the heat produced from tobacco heating can damage enamel by reducing salivary production and leading to more decay (Mori et al. 2023).

Both forms of tobacco consumption, while differing somewhat in their mechanisms and effects, have significant impacts on tooth structures and dental health in general. Understanding these complications is essential for developing effective preventive and treatment strategies. This study aims to evaluate the effect of CS and HT on the histological properties and discoloration of dental enamel and cementum. The null hypothesis assumed in this study states that CS and HT have the same impact on the ultrastructure or the color of human tooth enamel and cementum.

## 2 Materials and Methods

### 2.1 Specimens Preparation

This research project protocol was reviewed and approved by the Research and Ethics Committee of the Faculty of Dentistry, The British University in Egypt, with Project No. FD BUE REC 24‐001. Thirty intact and noncarious human maxillary premolars extracted for orthodontic treatment purposes, previously disinfected, were used. The sample size was determined using G‐Power software, and the test power of 80% and *a* = 0.5 were considered (Ibrahim and Hassan 2021). Crowns were separated from the roots (from the cementoenamel junction) to obtain enamel specimens. The buccal halves were sectioned using discs in a low‐speed handpiece under water cooling. Cementum specimens were obtained by sectioning the cervical parts of the roots (5‐mm length), then sectioned mesiodistally, and the buccal halves were used. No polishing was performed on either enamel or cementum surfaces. The specimens were then stored in distilled water at room temperature for 24 h. The specimens were randomly separated into six groups (*n* = 10), as follows: Group 1: enamel without smoking exposure; Group 2: enamel exposed to CS; Group 3: enamel exposed to HT; Group 4: cementum without smoking exposure; Group 5: cementum with CS exposure; and Group 6: cementum with HT exposure.

### 2.2 The Smoking Standardizing Apparatus

**Figure 1:**
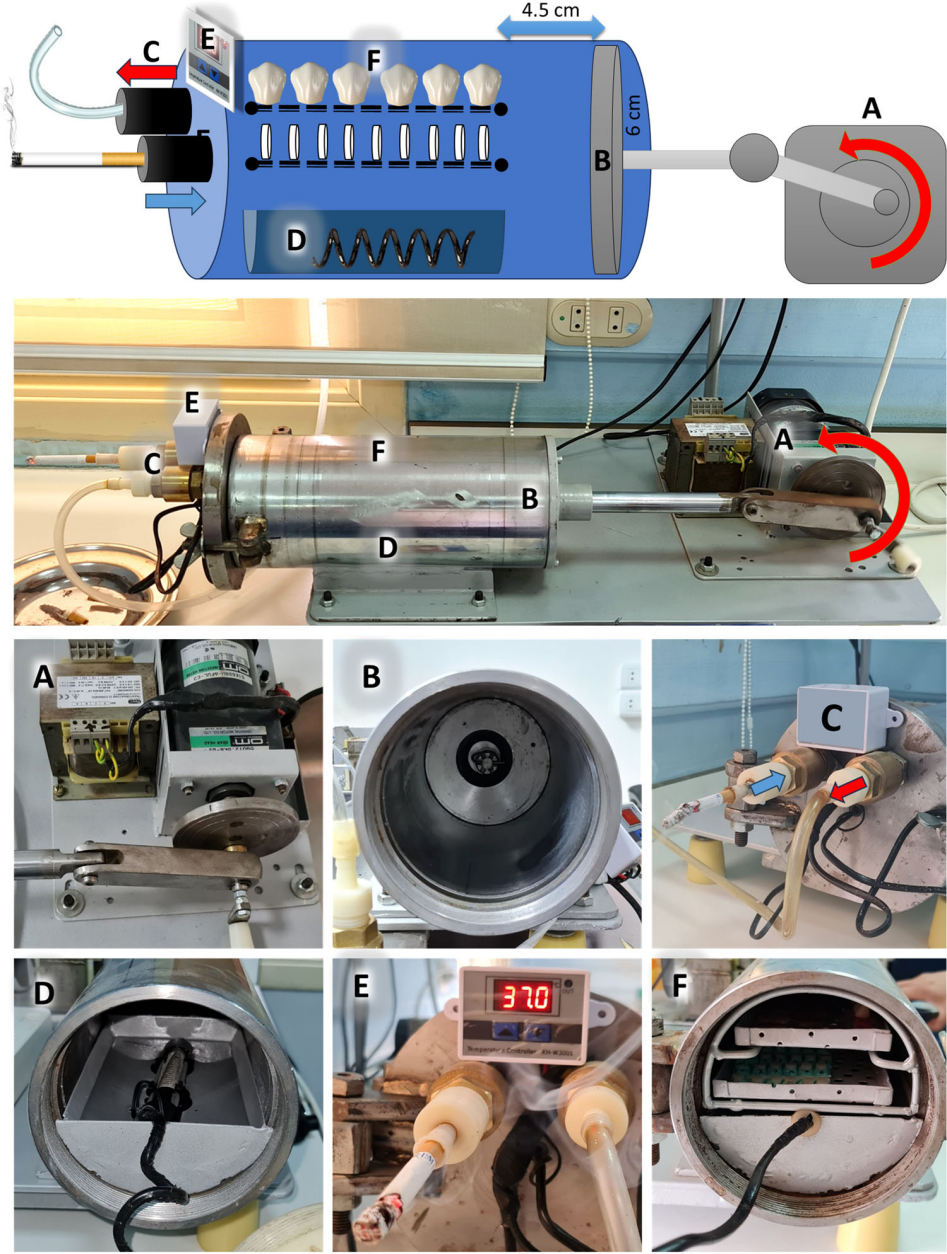
The smoking standardizing apparatus with a motor and gearbox with a crankshaft (A), a piston (B), two valves that allow inhalation and exhalation in one direction only (C), a pool of water with a heater (D) connected to a thermal sensor (E), and two perforated trays (F).

The smoking standardizing apparatus is specially designed for simulating the smoking process. It consists of a gearbox to reduce the speed of the motor to 2 Hz (2 cycles/s) with a crankshaft and connecting rod attached to a slider to change the rotation movement into linear movement (A) of 4.5‐cm length. Stainless steel cylinder with an internal diameter of 12 cm (6‐cm radius) with a piston (B) to generate a suction power with about 500‐mL volume (Sahu et al. 2013), simulating the tidal volume during smoking. A cigarette or electronic smoking device is attached to a valve that allows inhalation of the smoke in one direction only, simulating the mouth. Another valve only allows the exhalation in one direction, simulating the nose (C). A pool of water with a heater (D) connected to a thermal sensor regulates the temperature from 36.5°C to 37.5°C and 100% humidity, simulating the oral cavity (E). The samples (B) were mounted on two perforated trays to allow total exposure of all samples to smoke (F) (Figure 1). An additional movie file shows this in more detail (see Additional File 1).

### 2.3 Exposure of Specimens to Smoking

Conventional cigarettes (LM, Philip Morris International Inc., Egypt) and HT products (Heets, Russet selection, Philip Morris International Inc., Italy) were used in the experiments. The specimens were exposed to 600 cigarettes, representing 30 days of medium smoker behavior (20 cigarettes/day) (Shiffman 2009). Then, the specimens were gently washed with distilled water for 1 min, and the final color was measured with a spectrophotometer.

### 2.4 Color Measurement

Each specimen's three parameters of color were measured using a VITA Easyshade spectrophotometer Advance 4.01 (VITA Zahnfabric, Bad Sackingen, Germany) according to the CIE L*a*b* color order system. Mean measurements of the middle part of the middle one‐third of the buccal aspect were recorded before and after exposure of specimens to smoking. The color change (∆*E*) was calculated according to the following formula: Δ*E* = ([Δ*L*]^2^ + [Δ*a*]^2^ + [Δ*b*]^2^)1/2 (Shamel, Al‐Ankily, and Bakr 2019).

### 2.5 Scanning Electron Microscopy (SEM) and Energy‐Dispersive X‐Ray Spectroscopy (EDX) Analysis

Surface morphology was studied using images obtained from SEM of specimens from each group before and after the exposure of specimens to smoking. SEM (ThermoFisher (USA) Quattro S Felid Emission Gun, Environmental SEM “FEG ESEM”) at the Nanotechnology Research Center at The British University in Egypt was used to evaluate the surface topography and was analyzed using EDX at two different points to determine changes in surface chemical composition of calcium and phosphorus (Al Ankily et al. 2020).

### 2.6 Statistical Analysis

Statistical analysis of the obtained data was performed using SPSS for Windows (version 26.0; SPSS Inc., Chicago, IL, USA). The normal distribution of all variables was checked and verified using Kolmogorov–Smirnov and Shapiro–Wilk tests. The data were found to be normally distributed. Two‐way analysis of variance (ANOVA) followed by the post hoc Tukey test was used to assess the effect of CS and HT on the mean ∆*E* of the studied groups. Tukey's HSD post hoc test for multiple comparisons at a significance level (*α* = 0.05).

## 3 Results

### 3.1 Color Change

**Figure 2:**
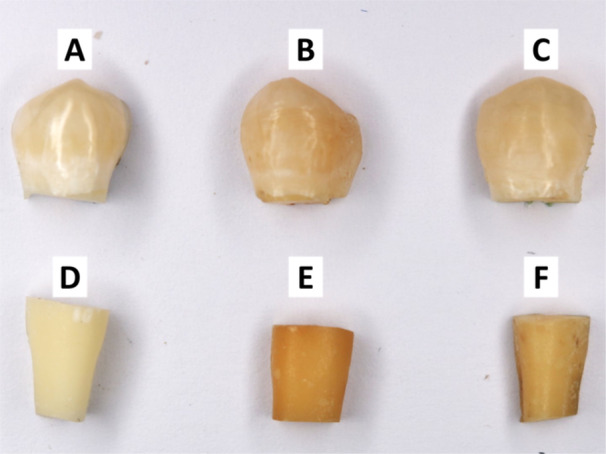
Naked eye color changes in different groups: (A) enamel control group, (B) cigarettes enamel group, (C) HT enamel group, (D) cementum control group, (E) cigarettes cementum group, and (F) HT cementum group.

**Figure 3:**
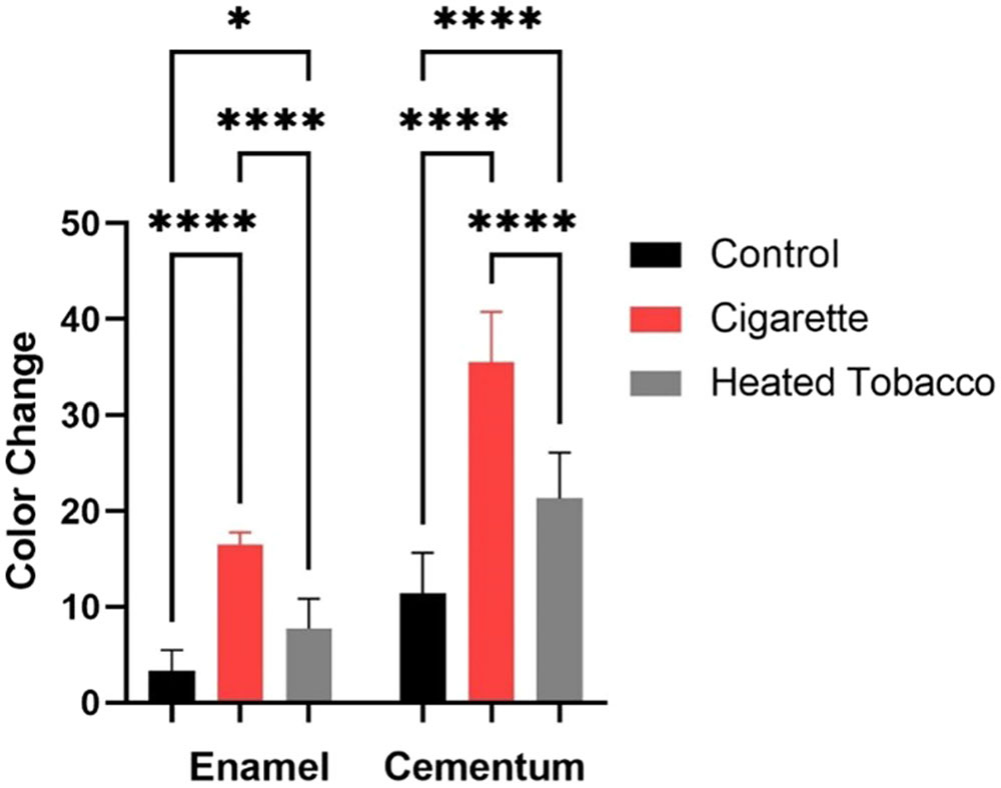
Statistical comparisons of color change (Δ*E*) measurements between groups. *p < 0.05 and ****p < 0.0001 indicate significance.

Results showed that CS had a more pronounced effect on enamel and cementum than HT (Figure 2). Summary and statistical comparisons of total color change (Δ*E*) measurements are shown in Table 1 and Figure 3. The Δ*E* caused by CS was statistically significant in enamel (*p* < 0.0001) and cementum (*p* < 0.0001) compared with the control group. Also, HT caused a significant difference in Δ*E* in enamel (*p* = 0.0322) and cementum (*p* < 0.0001) compared with the control group. Moreover, a significant difference was found when Δ*E* of CS and HT in enamel (*p* < 0.0001) and cementum (*p* < 0.0001) was compared. The impact of cigarette smoke and HT is more prominent in cementum than enamel.

**Table 1:** Means and standard deviations of Δ*E*.

	Control (mean ± SD)	Cigarette (mean ± SD)	Heated tobacco (mean ± SD)
Enamel	3.3 ± 2.1	16.5 ± 1.2	7.7 ± 3.1
Cementum	11.4 ± 4.1	35.5 ± 5.2	21.3 ± 4.7

### 3.2 SEM Analysis

**Figure 4:**
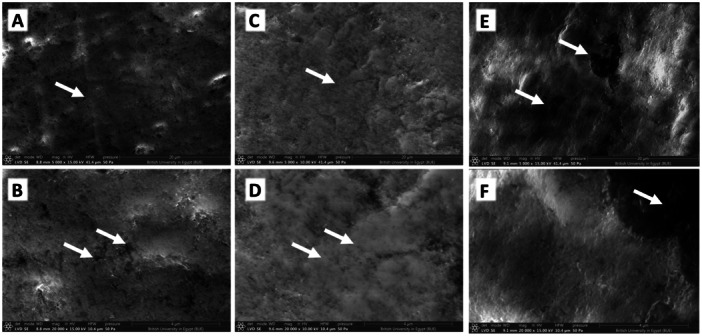
Scanning electron micrograph of the enamel surface of the control group (A) showing a rodless surface layer of enamel with some grooves and a few pores (arrows). Higher magnification reveals a nearly smooth surface with few grooves (B). Cigarette group (C, D) shows an enamel surface masked by an amorphous organic structure (arrows). The HT group (E, F) showed deep depressions and holes (arrows)—original magnification A, C, and E: ×5000, B, D, and F: ×20,000.

The SEM analysis of teeth not exposed to smoking revealed a mostly even and smooth outer layer of enamel, with occasional minor scratches, grooves, and a few pores (Figure 4A,B). On the other hand, the enamel surface of teeth exposed to CS is masked by an amorphous organic structure with some areas where the enamel rods were exposed and in other areas appeared destructed (Figure 4C,D). Teeth subjected to HT showed enamel surfaces with large and deep depressions (Figure 4E,F).

**Figure 5:**
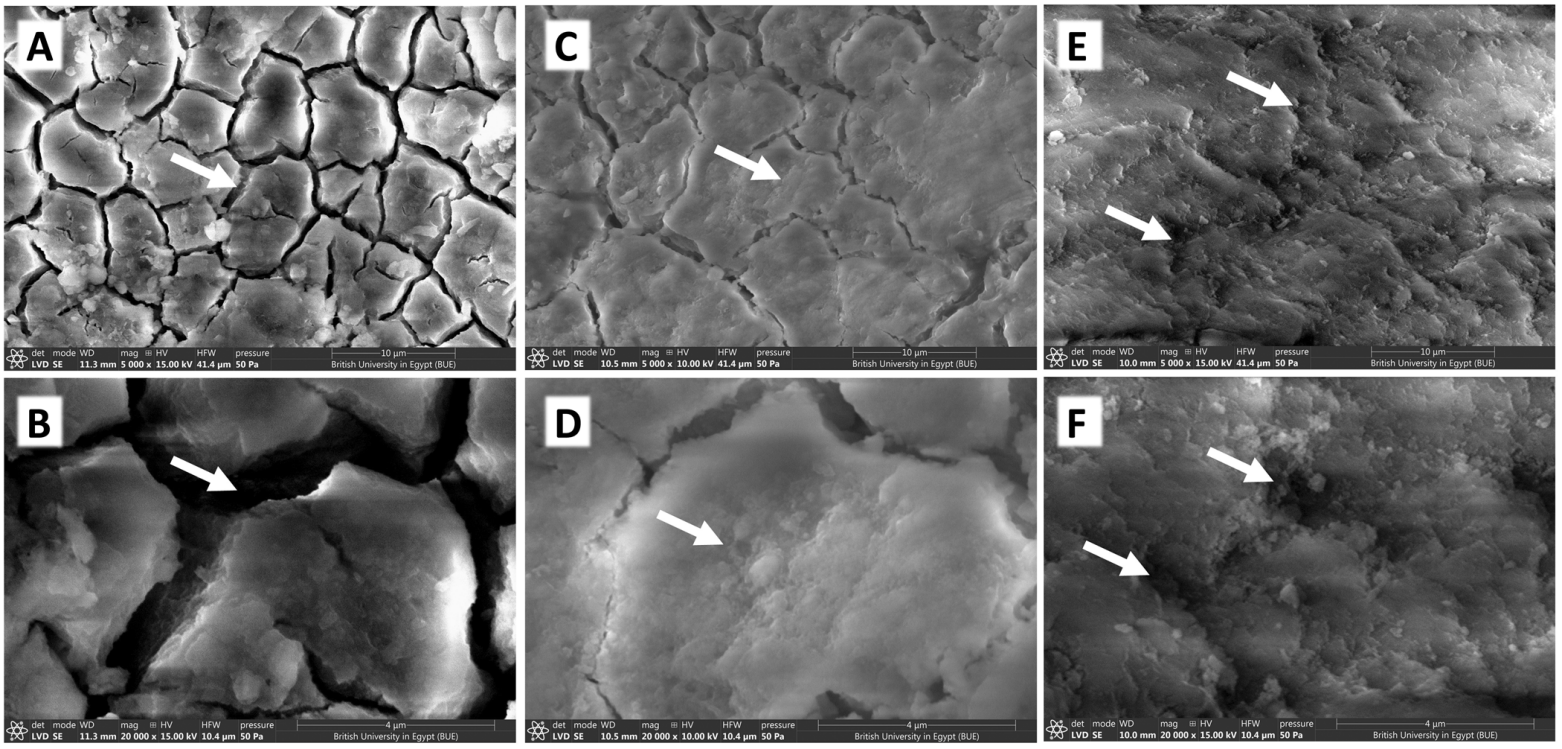
Scanning electron micrograph of cementum surface of the control group (A, B) shows regular, mosaic, and electro surface layer of cementum with regular and uniform grooves (arrows). Cigarette group (C, D) shows cementum surface masked by an amorphous organic structure (arrows) with irregular grooves. The HT group showed total loss of mosaic appearance and deep depressions (arrows) (E, F)—original magnification A, C, and E: ×5000, B, D, and F: ×20,000.

SEM evaluation of the cementum of teeth not exposed to smoking showed a surface layer of cementum with regular and uniform grooves (Figure 5A,B). The cementum of CS showed an amorphous organic structure with irregular grooves (Figure 5C,D), whereas HT caused a total loss of mosaic appearance and deep depressions (Figure 5E,F).

### 3.3 EDX Analysis

#### 3.3.1 Calcium Content

**Figure 6:**
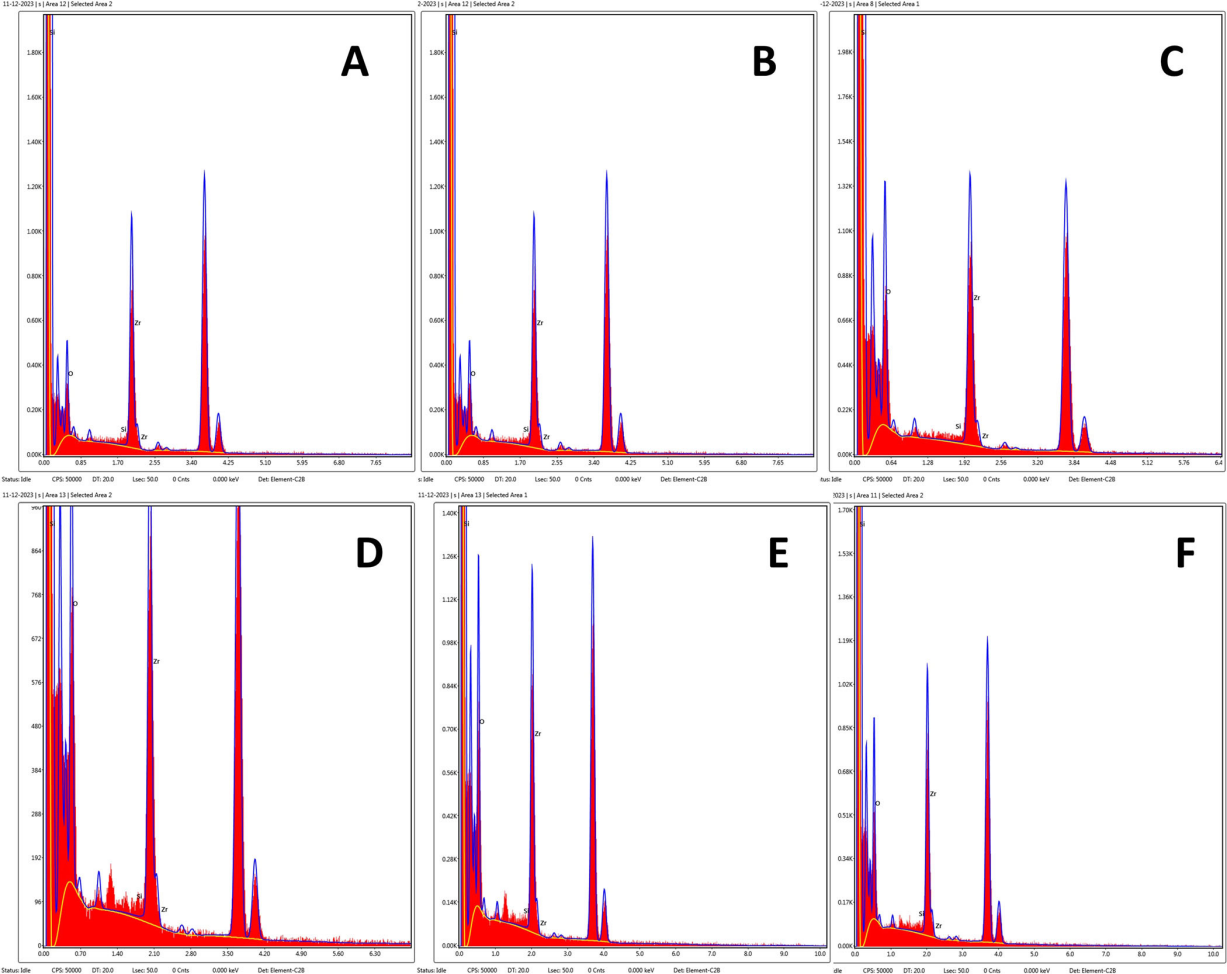
EDX graph of selected elements in different groups: (A) enamel control group, (B) cigarettes enamel group, (C) HT enamel group, (D) cementum control group, (E) cigarettes cementum group, and (F) HT cementum group.

**Figure 7:**
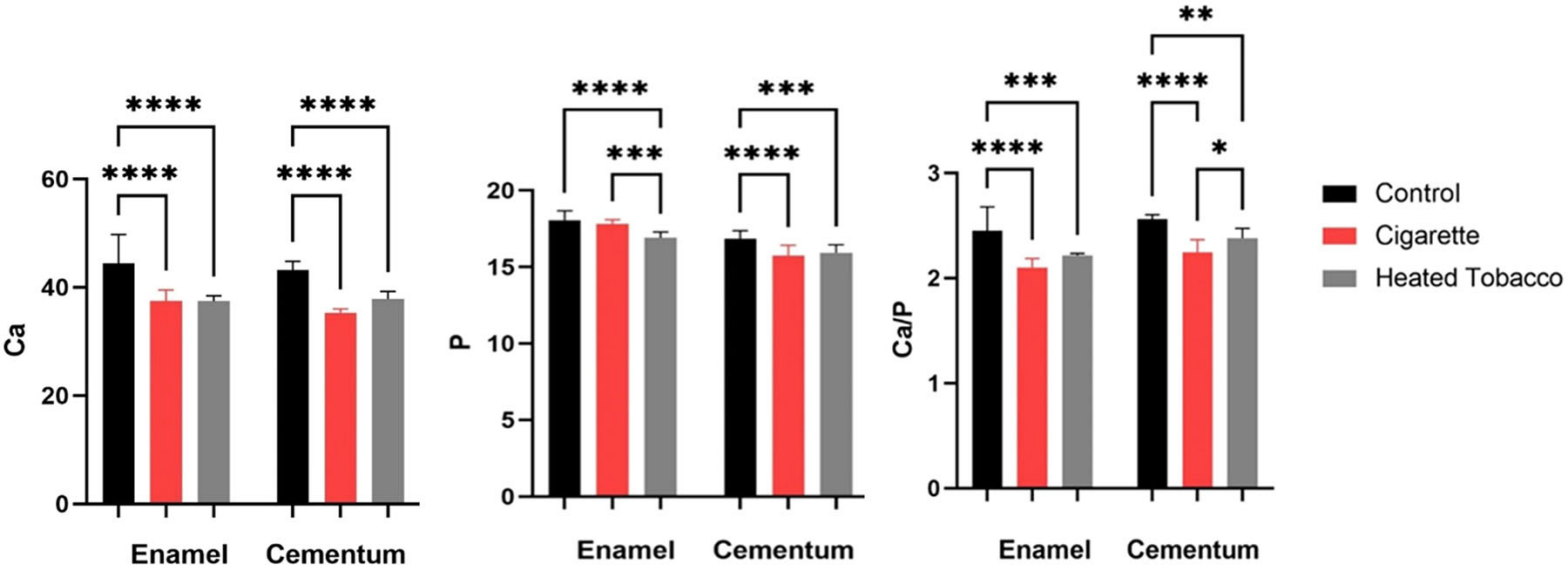
Statistical comparisons of calcium and phosphorus content and calcium/phosphorus ratio. Between groups. *p < 0.05, **p < 0.01, ***p < 0.001, and ****p < 0.0001 indicate significance.

CS and HT decreased calcium content for enamel and cementum compared with the control group. The reduction in calcium levels is statistically significant in enamel when comparing CS (*p* < 0.0001) and HT (*p* < 0.0001) with the control group. Similarly, there is a substantial decrease in calcium content in cementum (*p* < 0.0001). In enamel, the reduction in calcium content due to cigarette and HT exposure seems to be very similar, and a nonsignificant difference (*p* > 0.999) was found on comparing both groups. Similarly, cigarette exposure in cementum leads to a greater reduction in calcium content than HT exposure. However, this difference was insignificant (*p* = 0.08). Table 2 and Figures 6, 7 summarize the calcium content and statistical comparisons.

**Table 2:** Means and standard deviations of calcium and phosphorus content and calcium/phosphorus ratio.

	Control (mean ± SD)	Cigarette (mean ± SD)	Heated tobacco (mean ± SD)
Calcium			
Enamel	44.3 ± 5.3	37.4 ± 2.0	37.4 ± 1.0
Cementum	43.1 ± 1.6	35.3 ± 0.7	37.8 ± 1.0
Phosphorus			
Enamel	18 ± 0.6	17.8 ± 0.2	16.9 ± 0.3
Cementum	16.8 ± 0.5	15.7 ± 0.6	15.9 ± 0.5
Ca/P ratio			
Enamel	2.4 ± 0.2	2.1 ± 0.08	2.2 ± 0.01
Cementum	2.5 ± 0.04	2.2 ± 0.1	2.3 ± 0.09

#### 3.3.2 Phosphorus Content

CS and HT lead to decreased phosphorus content for enamel and cementum compared with the control group. In enamel, the reduction in phosphorus content due to cigarettes was insignificant (*p* = 0.57) compared with the control group, while a significant (*p* < 0.0001) decrease was found in HT exposure. HT showed a significant decline (*p* = 0.0006) in phosphorus levels compared with CS. CS exposure in cementum leads to a greater reduction in phosphorus content than HT. However, this difference was insignificant (*p* = 0.74). Table 2 and Figures 6 and 7 summarize the phosphorus content and statistical comparisons.

#### 3.3.3 Ca/P Ratio

When compared with the control group, the Ca/P ratio was significantly reduced (*p* < 0.01) in the enamel and cementum of CS and HT groups. In enamel, the ratio was not significantly (*p* = 0.09) reduced in CS when compared to HT. CS exposure in cementum leads to a more significant reduction in Ca/P ratio than HT. This difference was significant (*p* = 0.04). Table 2 and Figure 6 show the summary and statistical comparisons of phosphorus content.

## 4 Discussion

CS is a major contributor to preventable diseases and early death on a worldwide scale (Pan et al. 2019; Samet 2013). Internationally, the consumption of tobacco has emerged as a growing issue of public health significance (Perez‐Warnisher, De Miguel, and Seijo 2018). HT products are designed to deliver nicotine as an alternative to conventional cigarettes. HT products are marketed to customers as a comparatively less detrimental option to conventional cigarettes, benefiting both users and those nearby (Znyk, Jurewicz, and Kaleta 2021). The precise impacts of HT on the well‐being of individuals and its broader implications for public health remain primarily uncertain.

Prior studies have yet to thoroughly examine the relative effects of CS and HT on the composition and characteristics of enamel and cementum in human teeth. This study aims to investigate and compare the impact of CS and HT usage on the alteration of color, mineral composition, and structural modifications in the enamel and cementum of human teeth. To simulate real‐life conditions, a smoking device was fabricated, and smoking was performed to simulate medium smokers' habits of 20 cigarettes/day (Shiffman 2009) for 30 days.

Results of color change (Δ*E*) in the present study showed that both types of smoking had a significant (*p* < 0.05) effect on Δ*E* of enamel and cementum. These results agree with several studies indicating the impact of CS and HT on teeth color. The discoloration caused by smoking is attributed to the chemical composition of these products (Gasmi Benahmed et al. 2022). The presence of tar, nicotine, and erosive chemicals contributes to the discoloration. Moreover, previous studies showed that the heat produced during smoking is a major factor in the formation of stains on tooth surfaces (Gordon et al. 2022). Moreover, in the current study, CS caused a more significant (*p* < 0.05) Δ*E* than HT. CS has higher levels of harmful components in comparison with HT. In addition, the heat produced due to HT is much less than that of CS, leading to a less erosive effect on the surface of teeth and thus resulting in less discoloration (Haiduc et al. 2020). Furthermore, the reduced potential of staining for HT was attributed to the higher aerosols generated compared with cigarette smoke (Kurachi et al. 2023). This agrees with a study that revealed CS had a more prominent effect on discoloration of artificial denture teeth when compared with HT (Wang et al. 2022). This finding was extremely important to fill the current literature and knowledge gaps. Recent systematic reviews and meta‐analyses concluded that there was limited evidence supporting that electronic cigarettes and HT caused less intense dental staining and restoration on natural teeth than conventional tobacco products (Karanjkar et al. 2023; Paolone et al. 2023). This finding is of clinical significance as it informs clinicians to educate their patients about the potential stains caused by HT and the ability to remove those stains with polishing and whitening procedures should they happen.

On the other hand, the effect of CS and HT on discoloration was more pronounced in cementum than in enamel. This difference is due to the different composition of cementum, which is more porous than enamel, making it more prone to discoloration caused by smoking. Although the visual effect of discoloration of cementum is not as crucial as enamel, it may indicate the overall health and integrity of the supporting structures of the teeth (Sarna‐Boś et al. 2023, 2022). Furthermore, the detrimental consequences of cementum can significantly affect the stability of teeth since the debilitation of this region can jeopardize the periodontal status of teeth (Yamamoto et al. 2016).

SEM in the present study showed that CS and HT caused alterations in enamel and cementum morphological features. Enamel showed areas of destructed enamel rods and pores, which was more prominent in CS than HT. Previous studies documented comparable results where rods had a distinct breakdown with a collapse of their cores following exposure to CS (Ibrahim and Hassan 2021). During CS, the chemical substances emitted by cigarette smoke and the high temperatures produced by tobacco burning and combustion, ranging from 600°C to 1000°C, inevitably come into contact with dental enamel. Thus, in the present investigation, the observed alterations in the cellular structure caused by smoking can be attributed to these two primary concurrent processes. Moreover, exposing enamel to temperatures ranging from 200°C to 600°C has degraded the chemical structure, organization, and morphology of hydroxyapatite (HA) crystals in enamel (Mejía et al. 2016). This leads to the formation of pores, which increase the surface area and make enamel more permeable to acids. Similar effects were reported with regard to the surface roughness of 3D‐printed, CAD/CAM–milled, and conventional denture base materials (Mugri et al. 2023).

EDX analysis results revealed a reduction in calcium and phosphorus mass percentage. The primary inorganic constituent in dental enamel and cementum is HA, a mineral of calcium and phosphate ions. This drop in mineral content resulted in a lower Ca/P ratio, observed after the samples underwent demineralization due to mineral loss from the enamel and cementum caused by CS and HT exposure. Evaluating the Ca/P ratio has been demonstrated by numerous research as an effective method for determining the condition of tooth structures. Maintaining enamel and cementum's structural integrity and hardness requires a precise equilibrium between calcium and phosphorus (Sarna‐Boś et al. 2022). Any modification in this equilibrium can render enamel and cementum more vulnerable to harm and deterioration. Multiple studies have indicated that a reduction in the calcium‐to‐phosphorus ratio in enamel is associated with a loss in hardness, impairing enamel's functionality. Moreover, according to reports, the hardness of cementum is correlated with its Ca/P ratio. Root resorption is also associated with reducing this ratio (Yamaguchi et al. 2016; Yao‐Umezawa et al. 2017).

To correlate the results of the color changes and the histological analyses, changes in the enamel and cementum's crystalline structure and mineral content due to CS and HT could also contribute to its increased susceptibility to discoloration. A direct relation is found as the mineral levels decline and the crystalline structure is disturbed; the teeth become increasingly susceptible to surface discoloration caused by tar, nicotine, and other constituents of tobacco smoke. Furthermore, the microstructural damage resulting from demineralization creates a rougher and more porous surface, which not only compromises the mechanical strength of the teeth but also enhances the adherence of pigments and other staining substances. This correlation can elucidate the relationship between surface discoloration and underlying structural alterations in the enamel and cementum. This relationship has noteworthy clinical implications. It implies that measures targeting the prevention or reversal of the structural harm induced by tobacco smoking may also decrease the likelihood of discoloration.

The current results indicate that both CS and HT had a demineralizing effect on hard dental tissues. However, HT led to a significant reduction in the phosphorus content of enamel when compared with CS. At the same time, CS exposure in cementum resulted in a more significant decrease in Ca/P ratio than HT. Recent studies indicate that HT products and electronic cigarettes can significantly impair dental health by reducing fibroblast migration, worsening peri‐implant disease outcomes, and exacerbating inflammatory signs of periodontitis compared with conventional cigarettes. These effects are linked to increased pro‐inflammatory cytokines and harmful alterations in the oral microbiome (AlJasser et al. 2021; AlQobaly et al. 2022; Bamani et al. 2022; D'Ambrosio et al. 2022; Morishita et al. 2023; Thomas et al. 2022; Xu et al. 2022). Based on the studies discussed above, we hypothesize that the changes in the chemical composition of the coronal hard tissues were more significant in HT due to direct exposure to the excessive heat generated by HT compared with CS (Rad et al. 2010). On the other hand, the radicular hard dental tissues suffered from more mineral loss with CS as the harmful effects of HT seem to be more associated with the gingival and periodontal tissues as a result of the heightened inflammatory response, which appears to target mainly soft tissues at a cellular level, and to a lesser extent the hard dental tissues. Further research is necessary to confirm the correlation between the HT–associated inflammatory response and hard dental tissues.

## 5 Conclusions

CS and HT had similar effects on the surface morphology and the mineral content of teeth. However, CS demonstrated more significant color changes when compared with HT. It should be noted that the pattern of changes in the mineral content for each smoking method in the current study was site‐specific. HT decreased the mineral content of crowns, whereas CS had a significant effect on the mineral composition of the roots. The scope of this study might be restricted to observing the immediate effects of tobacco products on dental enamel and cementum, potentially omitting the long‐term impact that could provide a more comprehensive view of the consequences. A limitation of the study is that in a clinical setting, dietary habits and the presence of saliva with its mechanical washing action, antimicrobial properties, and buffering capacity could vary the effects of smoking between individuals. Additionally, this study does not account for oral hygiene practices, which could be a direction for future research.

## Author Contributions

Mahmoud Al Ankily designed the experiment and the smoking apparatus and reviewed the manuscript. Fatma Makkeyah performed the experiment and collected the data. Mahmoud M. Bakr wrote the manuscript and interpreted the data. Mohamed Shamel wrote the manuscript and performed the statistical analysis. All authors revised and approved the final manuscript.

## Ethics Statement

The study protocol was approved by the Research Ethics Committee of the Faculty of Dentistry, The British University in Egypt (No. FD BUE REC 24‐001).

## Consent

The authors have nothing to report.

## Conflicts of Interest

The authors declare no conflicts of interest.

## Supporting information

Supporting information.

## Data Availability

The data that support the findings of this study are available from the corresponding author upon reasonable request.
